# SARS-CoV-2 and HIV-1: So Different yet so Alike*.* Immune Response at the Cellular and Molecular Level

**DOI:** 10.7150/ijms.73134

**Published:** 2022-10-03

**Authors:** Catherine Demoliou, Christos Papaneophytou, Vicky Nicolaidou

**Affiliations:** Department of Life and Health Sciences, School of Sciences and Engineering, University of Nicosia, 46 Makedonitissas Avenue, 2417, Nicosia, Cyprus

**Keywords:** COVID-19, SARS-CoV-2, HIV-1, lymphopenia, immune response

## Abstract

In the past half century, humanity has experienced two devastating pandemics; the HIV-1 pandemic and the recent pandemic caused by SARS-CoV-2. Both emerged as zoonotic pathogens. Interestingly, SARS-CoV-2 has rapidly migrated all over the world in less than two years, much as HIV-1 did almost 40 years ago. Despite these two RNA viruses being different in their mode of transmission as well as the symptoms they generate, recent evidence suggests that they cause similar immune responses. In this mini review, we compare the molecular basis for CD4^+^ T cell lymphopenia and other effects on the immune system induced by SARS-CoV-2 and HIV-1 infections. We considered features of the host immune response that are shared with HIV-1 and could account for the lymphopenia and other immune effects observed in COVID-19. The information provided herein, may cast the virus-induced lymphopenia and cytokine storm associated with the acute SARS-CoV-2 infection and pathogenesis in a different light for further research on host immune responses. It can also provide opportunities for the identification of novel therapeutic targets for COVID-19. Furthermore, we provide some basic information to enable a comparative framework for considering the overlapping sets of immune responses caused by HIV-1 and SARS-CoV-2.

## Introduction

In the past half century, two distinct, novel RNA viruses have caused global pandemics, both having emerged as zoonotic pathogens: human immunodeficiency virus type 1 (HIV-1) and severe acute respiratory syndrome coronavirus 2 (SARS-CoV-2) 1. HIV-1, a member of the *Retroviridae* virus family, is most closely related to immunodeficiency viruses found in wild chimpanzees and most likely made the transfer to humans early in the 20^th^ century 2. HIV-1 is transmitted sexually or through body fluids, such as blood and breast milk, and leads to a chronic infection that eventually results in immunodeficiency and death by opportunistic infections.

Coronaviruses (CoVs), like the SARS-CoV-1 3, the Middle East Respiratory Syndrome Coronavirus (MERS-CoV) 4, and their recently isolated relative SARS-CoV-2 5, are all enveloped viruses with a closely related positive RNA genome, and zoonotic origin. The most recent member, SARS-CoV-2, is most closely related to a virus isolated from bats, it is transmitted via the respiratory route and has spread worldwide causing a pandemic 6. The WHO reports that to date there are more than 583 million confirmed cases of infection, including more than 6 million deaths of the related disease, Coronavirus disease 2019 (COVID-19) 7.

CoVs contain four major structural proteins, of which the Spike (S) protein is used for host receptor attachment and host cell entry, facilitated via membrane fusion^8^ and via priming by the Transmembrane serine protease 2 (TMPRSS2) 9. The host receptor is the cell membrane angiotensin-converting enzyme 2 (ACE2) receptor, which converts angiotensin II into angiotensin. The ACE2 receptor is an interferon (IFN)-stimulated gene, co-expressed with the serine protease TMPRSS2 in the upper airway human respiratory epithelial cells, human absorptive enterocytes, nasal goblet secretory cells 10 and vascular cells of many tissues, including the lungs, heart and kidney, and it contributes to the SARS-CoV-2 organotropism especially for patients with pre-existing conditions 11-14.

COVID-19 symptoms can range from asymptomatic to acute and may result even in death. In mild cases, viral infiltration of lung parenchymal tissue triggers an innate immune response. This is accompanied by the secretion of cytokines and chemokines 15, triggered by the viral RNA 16 due to the activation of pattern recognition receptors (PRRS), RIG-I-like receptors (RLRs) and the Toll-like receptors (TLRs) of Natural Killer (NK) cells and monocytes/macrophages of the host. If the innate response is compromised at the alveolar cell level, the virus has the chance to replicate, causing extensive tissue injury that dictates the severity of infection. In acute infection cases, the overproduction of pro-inflammatory cytokines, known as “cytokine storm”, driven by IL-6, CXCL10 and infiltrating macrophages 17-20, results in the suppression/deregulation of the immune response by T cells and in lymphopenia 21,22, as well as the acute respiratory distress syndrome (ARDS), which may lead to organ dysfunction, and death 23-25.

CoVs and HIV-1 belong to different viral families, differ significantly in their ecology, mode of infection, and genome. Another important difference is the fact that HIV-1 infection cannot be cleared, which constitutes a major challenge in eliminating the virus, something which is not an issue in SARS-CoV-2 infection. The two viruses do however, share important similarities; recent reviews have compared them in terms of their evolution 1. Similarities also exist regarding the immune responses caused by the two viruses.

**Figure 1:**
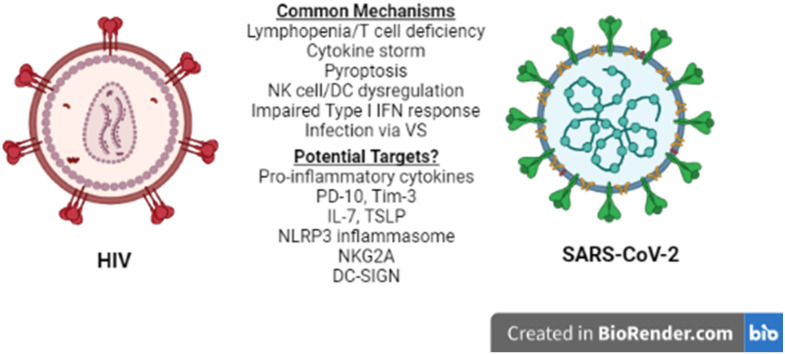
** HIV-1 vs SARS-CoV-2.** Similarities between HIV-1 and SARS-CoV-2 immune response to infection at the cellular level reveal common mechanisms and may lead to the identification of potential therapeutic targets for COVID-19. NK: Natural Killer, DC: Dendritic cell, VS: Virological Synapse.

Lymphopenia is associated with several other viral infections including HIV-1 26, and is considered a prognostic value parameter for individuals that are infected by SARS-CoV-2 or HIV-1. Although the manifestations of HIV-1 infection on the immune system become apparent in the chronically ill patients 26, they are driven by the cytokine profile set early in the disease and, therefore, several mechanisms and molecules proposed to play a role in acute HIV-1 lymphopenia may also be shared in the SARS-CoV-2-induced pathogenesis. A recent comparison of the pathologies and side effects of infection by these two viruses has highlighted the similarities in the pro-inflammatory cytokine response, the modification of intestinal microbiota and the formation of Neutrophil Extracellular Traps (NETs) 27. Understanding COVID-19-associated lymphopenia in the framework of HIV-1 infection may identify potential molecular targets for further research and/or development of treatment(s) for COVID-19 (**Figure 1**).

## T lymphocytes and granulocytes

HIV-1 and SARS-CoV-2 are different viruses. However, T lymphocyte deficiency or lymphocyte ineffectiveness and a “cytokine storm” due to the hyperinflammatory response of cells involved in the innate response, are characteristics of both 26-30. In the case of acute HIV-1 subtype B and C infections, the pro-inflammatory “cytokine storm” activation contributes to CD4^+^ T cell lymphopenia and prevention of IL-2 producing memory CD4^+^ and CD8^+^ T cells, which lead to the development of the acquired immune deficiency syndrome (AIDS). The majority of T cells die by pyroptosis, linking the HIV-1 infection and lymphopenia with dying CD4^+^ T cells releasing signals that cause more cells to die thus, preventing T cell homeostasis and renewal 31-33.

There are distinct differences in the numbers of blood cells like macrophages, CD8^+^ T cells, Th17 cells and naive T cells between HIV-1 and severe COVID-19 patients. However, in both types of infections, the common feature is that cells like NK cells, B cells, CD4^+^ T cells, regulatory T cells, memory T and B cells are reduced significantly in numbers 26. There is a reduction in CD8^+^ T cell observed in COVID-19, which is attributed to cell exhaustion due to continuous exposure to cytokines like IL-10, IL-6, and Tumor Necrosis Factor-alpha (TNF-α), to damage-associated molecular patterns and to virus-derived antigens 34. The cell cytokine profile resembles that of terminally exhausted CD8^+^ T cells with compromised cytotoxic activity, seen in patients chronically infected with HIV-1 35-37. Increased COVID-19 severity and chronic HIV-1 infection are accompanied by high expression of T cell inhibitory receptors including programmed cell death-1 (PD-10) and T cell immunoglobulin mucin 3 (Tim-3) proteins, which contribute to the low CD4^+^ T cell counts and reduction of IFN-γ production. This immunopathogenic late CD4^+^ T cell response is considered to provide potential targets for treating T cell exhaustion via inhibition of expression of these inhibitory receptors 36-39.

Recent studies in T cells infected with HIV-1 have indicated the role of restriction factors like APOBEC3G (apolipoprotein B mRNA editing enzyme, catalytic polypeptide-like 3G), Tetherin (CD317), SAMHD1 (SAM domain and HD domain-containing protein 1), and TRIM5α (Tripartite motif-containing protein 5,) in the front-line defense against HIV-1 infection. These factors are associated with the intracellular protein degradation pathways involved in viral antigens production and the activation of HIV-1-specific T cell immunity. HIV-1 has evolved the ability to counteract these by inhibiting their expression using several accessory viral proteins or evading through protein variability, and thus replicate efficiently in the hostile environment of the host cell contributing to T cell death 39,40. Functional proteomic atlases of HIV-1 and SARS-CoV-2 cell infections *in vitro* 41,42, have identified a large number of cellular host restriction factors against HIV-1 and SARS-CoV-2. Comparisons of these along with the atlas of phosphorylated proteins in SARS-CoV-2 infection 43 could provide valuable clues in the understanding of the mechanisms underlying viral inactivation at the transcriptional level, and help identify molecular targets and therapeutic or curative strategies for COVID-19 treatment.

Little is known about the “cytokine storm” contribution in HIV-1 or SARS-CoV-2 immunopathology of eosinophils, basophils and neutrophils. A positive correlation between neutrophil cell counts and cytokines, like IP-10 and IL-8, has been reported in acute HIV-1 infection 29. In COVID-19 cases, neutrophil and granulocyte pathophysiology has been linked to the adverse effects of SARS-CoV-2 on tissues, as well as the vascular and coagulation systems in severely affected patients 44. The increase in mature and immature neutrophil numbers and decrease in the numbers of eosinophils and basophils in severe COVID-19 infections, have been associated with altered expression of several receptors involved in activation, adhesion and migration of granulocytes (e.g. CD62L, CD11a/b, CD69, CD63, CXCR4) 45. As in the case of acute HIV-1 infection 29 and HIV-1 dissemination to CD4^+^ T cells 46, identifying SARS-CoV-2 role in the expression of the chemokines/chemokine receptors responsible for mononuclear cell trafficking and tissue infiltration observed in post-mortem analysis of COVID-19 patients 47, will be important. These routes of infection (i.e. blood monocytes) could explain the persistent tissue infectivity of the SARS-CoVs and contribute to the persistent inflammation caused by the antibody-dependent enhancement (ADE) mechanism, a viral dependent phenomenon observed in patients who remain virus positive although they have early, suboptimal antibody activity 21.

Alternatively, identifying upstream regulators of the inflammatory pathway, as for example IL-17 studied in mice 48, or regulators of lymphopoiesis, as for example IL-7 and thymic stromal lymphopoietin (TSLP), may be needed to understand COVID-19 cytokine pathogenesis. IL-7 is produced by intestinal epithelial and epithelial goblet cells and plays an important role in the development, proliferation, and survival of innate and adaptive lymphoid cells as well as in the development of the multiple effector functions during infections 49,50. IL-7 was used in HIV-1 infections to increase *de novo* T cell formation but led to rapid proliferation of the latent HIV-1 reservoir in resting memory CD4^+^ T cells, and any further treatment was abandoned 28. IL-7, however, may be of use in the treatment of severely ill COVID-19 patients to counterbalance lymphopenia and enhance antiviral response of the immune system 51.

TSLP is produced by epithelial cells of the skin, gut and lungs and it is considered to play a critical role in driving Th2 mediated inflammation, and to contribute to asthma and allergic inflammation 52. TSLP expression is activated by cytokines including IL-4, IL-1 and TNF-α 53, and high TSLP expression appears to play a role in platelet activation and thrombosis in Kawasaki disease 54, as well as in the metabolic syndrome and high blood pressure of severely obese individuals 52-55. HIV-1-induced expression of TSLP in epithelial cells triggers dendritic cell (DCs)-mediated amplification of HIV-1 infection in activated CD4^+^ T cells 56. TSLP, therefore, could be a good candidate for further investigations in relation to SARS-CoV-2 infection and related side effects. TSLP and its isoforms may contribute to the Kawasaki Syndrome seen in SARS-CoV-2 infected children, or to increased thrombosis in COVID-19 severe cases, through activation of vascular endothelial cells and/or platelet activation via TSLP-dependent PI3K/Akt signaling 57-59.

It has been well documented that the lymphopenia in acute SARS-CoV-2 infection is the result of tissue damage inflammation and a dysregulated innate and adaptive immune system that involves a dramatic loss of CD4^+^ and an even greater loss of CD8^+^ T cells 60, 61. Since T cell infection by SARS-CoV-2 is abortive 62, tissue destruction and cell death have been attributed to cytokine induced tissue necrosis and apoptosis and to T cell driven pyroptosis via inflammasomes activation, rather than viral replication 63, 64. Pyroptosis-driven CD4^+^ T cell death following SARS-CoV-2 infection 65, 66 is supported by the increased serum levels of IL-1β and IL-18 in COVID-19 patients. Pyroptosis occurs faster than apoptosis and the release of cell contents results in the recruitment of increasing numbers of effector immune cells, thus promoting further the inflammatory cascade and T cell exhaustion 63, 66-69. In HIV-1 acute infection, studies have shown that more than 95% of CD4^+^ T cells depleted from lymphoid tissue die by pyroptosis due to abortive HIV-1 infection and inefficient reverse transcription 31, 68. Pyroptosis is also involved in CD4^+^ T cell loss in chronically HIV-1-infected patients via the NLR family pyrin domain containing 3 (NLRP3) sensor in both peripheral blood and lymphoid tissues in a bystander manner 70. HIV-1 induced CD4^+^ T cell death by pyroptosis is not inhibited by antibodies to IFN α/β, suggesting that pyroptosis is not normally part of the innate immune responses that promote cell death in acute infections. Additionally, caspase-3 expressing cells in HIV-1 productively infected cells, appear to be anatomically separated from cells with abortive infections 63, 68. Targeting pyroptosis could potentially be a beneficial approach to address lymphopenia in COVID-19 62, 71, and implicate pyroptosis signaling as a target for anti-HIV-1 treatment.

## Natural Killer Cells and CD8^+^ Cytotoxic T Cells

NK cells along with CD8^+^ cytotoxic T cells are central to the host defense against viral infection and tumour cells. NK cells recognize and kill virally-infected and neoplastic cells directly or via antibody dependent cellular cytotoxicity (ADCC), using the granzyme-perforin system that leads to apoptosis. NK cells are also able to kill via cytokine release (i.e. TNF) and expression of ligands such as FasL and the Tumor necrosis factor-related apoptosis-inducing ligand (TRAIL). NK cells have additionally an immunoregulatory role via secretion of and response to various cytokines (IFNs, IL-12, -15 and -18), which prime them to respond to viral infection via IFN-γ production and/or NK cell proliferation 72, 73. NK cells are also activated by stress induced ligands via their cell surface lectin receptors like CD94/NKG2C, and express inhibitory receptors, like CD94/NKG2A, to repress NK cell activation and cytotoxicity. NKG2A positive NK cells, therefore, play an important role in limiting excessive activation, preventing apoptosis, and preserving CD8^+^ T cell responses during viral infections 73-75.

Upon chronic cytokine stimulation there is exhaustion of adaptive NK cells, associated with down modulation of CD94/NKG2C receptor expression via epigenetic reprograming, which is prevented through signaling involving the NKG2A inhibitory receptors 76. NK cells from HIV-1 infected individuals have increased- whereas seronegative individuals have decreased-NKG2A receptors due to changes in cell phenotype and exhaustion. High levels of HIV-1 replication alters the NK cell phenotype and promotes the expansion of an anergic subset, unable to perform ADCC and to kill virus infected CD4^+^ T cells. The reduced numbers of cytotoxic NK cells and higher NKG2A expression in patients with late-stage HIV-1 infection were related to the escape of HIV-1-infected CD4^+^ T cells from NK cells cytotoxicity 77, 78.

A similar dysregulation of NK cell expression of the NKG2A inhibitory receptor due to SARS-CoV-2 induced hyperinflammation could also lead to NK cells exhaustion with diminished capacity to produce TNF and IFN-γ cytokines, as in the case of HIV-1 infected cells 76, 79. The decreased numbers of NK and CD8^+^ T cells have been positively related to the severity of COVID-19, to the increased expression of NKG2A inhibitory receptors, and to the reduced expression of lysosomal-associated membrane protein-1, IFN-γ, IL-2, granzyme B, and TNF-α (normally present in chronic viral infections). In contrast, NKG2A expression was reduced in patients recovering from SARS-CoV-2 infection, suggesting an early functional exhaustion of cytotoxic lymphocytes 30, 80, 81. Furthermore, SP1 expression in lung epithelial cells was shown to contribute to the exhaustion of NK cells via HLA-E/NKG2A interactions 80. It is of interest to note that patients who recover from COVID-19 have SARS-CoV-2 specific CD8^+^ and CD4^+^ T cells 82, supporting the fact that, the SARS-CoV-2 virus may have mechanisms that evade the efficient innate immune response 83. Taken together, these findings highlight the importance of improving the early immune response of NK cells in addition to cytotoxic T cells to prevent their exhaustion, and that the NKG2A receptor in NK cells could be a potential target to enable virus elimination in the early stage of SARS-CoV-2 infection.

## Dendritic cells

Antigen presentation to T cells is a key event for their activation, which requires the contribution of mature DCs, the immune cells that are considered as the link between the innate and adaptive immune systems. HIV-1 infection of DCs prevents DC maturation, which results in their continuous stimulation and elimination of infected T cells, enhancement of central memory CD4^+^ T cell proliferation and differentiation, leading to exhaustion of the CD4^+^ T cell pool, while at the same time generating increased target cell numbers for HIV-1 infection. The secreted type I IFNs also induce the expression of immunosuppressive molecules like PD-L1 (a suppressor of the adaptive immune response as discussed earlier) at the surface of DCs, leading to diminished anti-viral responses 84.

Studies on DCs infected with SARS-CoV-1, concluded that although infection could be abortive, the virus negatively modulated the DC expression of antiviral cytokines and promoted death receptor ligands and chemokine receptors. This facilitated the migration of cytotoxic DCs from the infection site to lymph nodes of SARS-CoV-1 infected patients for further depletion of lymphoid cell through TRAIL induced apoptosis 85-87.

At lymph nodes and peripheral tissue, infected DCs may promote SARS-CoV-2 infection of T cells through virological synapses (VS), in a manner similar to that of HIV-1 infected DCs 88, 89. VS, (i.e. cell to cell transfer), is used by a number of intracellular pathogens and viruses in order to spread through their host 90. More interestingly, studies on HIV-1 transmission have shown DCs participation in CD4^+^ T cell trans-infection, despite the low infectivity of DCs. This involves the virus attachment to C-type lectins, such as the DC‐specific intercellular adhesion molecule‐3‐grabbing nonintegrin (DC-SIGN). Attachment is followed by endocytosis, storage and viral dissemination through the exosome pathway, or through an “infectious synapse” between uninfected mature DCs harboring HIV-1 and uninfected target cells with retention of the virus on the DC surface and transmission to target cell through adhesion protein interactions. DCs were reported to take up SARS-CoV-1 via the DC‐SIGN-receptor and transfer the virus to uninfected target cells through direct cell-to-cell transmission, suggesting that the CoVs family of viruses may also infect T cells “in trans” via VS 91, 92. DC-SING and DC-SING receptor (DC-SINR) enhance SARS-CoV-1 and SARS-CoV-2 S protein-driven transinfection 93, 94, and there is evidence that DC-SINGR expression is induced in alveolar macrophages by IL-4 and IL-13 upon infection by other viruses 95. Since the levels of these cytokines increase in the early phases of SARS-CoV-2 infection, they could also be involved in macrophage infectivity at lymph node sites. Lymphopenia, in COVID-19, in addition to innate response 60 may also occur via transmission via DC-T cell VS, T cell-T cell VS and even macrophage-T cell VS infection with SARS-CoV-2, in a manner similar to that of HIV-1 transmission 92, 94. It is of interest to note that HIV-1 taken up via DC-SIGN remains infectious for prolonged periods of time, protected possibly by trafficking through non-lysosomal endosomes and infecting T cells through DC-mediated viral transmission 89. DCs' role in the transmission of SARS-CoV-2 virus, T cell dysregulation and functional exhaustion could provide targets for development of therapeutic approaches.

## Macrophages

The acute onset of lung inflammation is key to ARDS present in severe COVID-19 cases and mortality. Alveolar and lung interstitial macrophages have a central role in SARS-CoVs infection and ARDS since they contribute to the “cytokine storm” through massive production of cytokines, monocyte chemoattractant proteins, nitric oxide (NO) and reactive oxygen species (ROS) 17, 58, 96, 97. This cytokine storm may also be followed by a secondary haemophagocytic lymphohistiocytosis (sHLH) response, a hyperinflammatory syndrome, and more likely result in fatal acute lung injury and multi-organ failure 98.

Early timing of type I IFN production by activated macrophages, appears to prevent the lethal inflammatory cytokine storm 99, 100. The sera from critically ill patients who died of SARS promoted the accumulation of pulmonary proinflammatory macrophages with enhanced SARS-CoV-1-induced MCP1 and IL-8 production, and this was counteracted by the blockade of FcγR 101. It has therefore been considered, that macrophage activation by SARS-CoV-1 could be mediated by antibody (anti-S-IgG) dependent enhancement (ADE) via the engagement of Fc receptors (FcR) 102, 103. It has also been suggested that ADE may facilitate the spread of SARS-CoV-2 in infected hosts, although *in vivo* evidence from human studies is still required for further support regarding this path of infection 104. However, internalization of virus-antibody immune complexes through the FcRs can upregulate pro-inflammatory cytokines, downregulate anti-inflammatory cytokines and induce cell death of recruited immune cells infected with SARS-CoVs either through ADE or the ACE2 receptor and/or through VS 69, 105.

In the case of HIV-1, non-dividing, metabolically active macrophages have been shown to contain large quantities of biologically active HIV-1 DNA, which is unintegrated and can remain stable and contribute to HIV-1 pathogenesis 106. HIV-1-infected macrophages can engage motile T cells in stable contacts through binding of virus- and host-derived adhesive molecules and contribute to high viral spread and HIV-1 progression 107. Using a similar path of dissemination, SARS-CoV-2 may also persist in resident macrophages of many tissues after recovery from COVID-19, since they are differentiated cells and have a long life span. GU-rich RNA derived from SARS-CoV-2, SARS-CoV-1, and HIV-1 have been shown to induce cytokine release (IL-6, TNF, and IL-1b) from human primary macrophages in the absence of viral infection, associating SARS-CoV-2 and HIV-1 to chronic inflammation 69. The presence of infected macrophages at lymph nodes and their tissue redistribution may prevent the elimination of the virus and explain how a small percentage of patients recovering from SARS-CoV-2 may become positive again for SARS-CoV-2 RNA, and/or be characterized as virus carriers 108, 109.

## Conclusions

COVID-19 is multiphasic in its pathogenesis, and includes a cytokine storm, lymphopenia and ARDS, which indicate poor prognosis. COVID-19 progression and severity are associated with a systemic dysregulation of the immune system and impaired type I IFN early responses. Tissue damage and signatures of early innate antiviral responses by lymphocyte and monocyte infiltration and macrophage accumulation in the infected alveolar lumen, are all markers of ARDS. The death of infected monocytes/macrophages, NK cell activation, NK cell-mediated ADCC, NK cell and T cell dysregulation and exhaustion are considered to be the contributors of proinflammatory cytokines and to drive the cytokine storm and lymphopenia. Although lymphopenia is observed in many viral infections, it is poorly understood and more so in the case of COVID-19, where the immune cell dynamics and those of cytokine secretion remain unclear. Furthermore, the evidence for T cell death due to direct infection by SARS-CoV-2 is insufficient and needs to be confirmed.

Comparative studies on immune cell dysfunction in SARS-CoV-1, MERS-CoV and HIV-1 infection, may help in understanding the pathogenesis of SARS-CoV-2 and identifying important components for further research. Common mechanisms highlighted in this review are summarized in Table 1. Infection of T lymphocytes by SARS-CoV-2 via VS in a manner similar to that used by HIV-1 for its dissemination in the host, although abortive, could contribute to lymphopenia due to virus-induced pyroptosis. Furthermore, SARS-CoV-2 infection and the systemic hyperinflammatory response may cause a state of T cell exhaustion with effector functions that may contribute via cytokine secretion and cytotoxicity to COVID-19 lymphopenia and pathogenesis. Such processes would justify strategies aiming the blockade of cytokine activity and cytokine receptor signaling at the early stages of COVID-19 using anti-inflammatory medication. Effective drug design and targeting of specific cytokines and their receptors, however, may first require a better understanding of the crosstalk and interactions of the pathways activated by these cytokines, as well as the stage specific factors that contribute to the pathology of SARS-CoV-2 infection.

## Figures and Tables

**Table 1:** Common mechanisms affecting immunity in HIV-1 and SARS-CoV-2 infection.

Common molecular/cellular mechanisms	Associated immunological/pathological effect	Reference
**Cytokine storm/ inflammasome activation**	T cell lymphopenia due to death by pyroptosis T cell exhaustion Immunodeficiency	(26-34)
**Elevated T cell inhibitory receptors**	T cell lymphopenia Reduced I IFN-γ production	(36-39)
**Altered host restriction factors**	Inability to control viral infection	(41,42)
**NK cell dysregulation**	Reduced cytokine/inhibitory receptor expression NK cell exhaustion, inability to kill virally infected cells	(76-79)
**Dysregulated and infected DCs**	Reduced T cell activation Viral spreading via VS	(84-89) (91-94)
**Impaired Type I IFN response**	Inability to control viral infection	(76,79)
**Viral persistence in macrophages**	Production of pro-inflammatory cytokines Viral spreading	(107-109)
